# Syphilitic Lesions of the Osseous System in Infants

**Published:** 1875-06

**Authors:** 


*Syphilitic Lesions of the Osseous System in Infants and Young Children.* By R. W. Taylor, M. D. New York: Wm. Wood & Co., 1875. Buffalo: H. H. Otis.

This work possesses the rare merit of being based almost wholly upon personal experience and investigation, and may be considered as representing alone the the views of the author. Dr. Taylor tells us, that when his attention was first drawn to the lesions of syphilitic nature occurring in young children, he found that he was obliged to study the disease itself, as but few works mention it, and those only in a casual manner. In order to show the essential differences between scrofulous and rickety affections and those of a specific nature, the author was obliged to go into a close analysis of the cases, and in order to establish a new feature in diagnosis, he found it necessary to dwell to a considerable extent upon the separation of the epiphysis, which depends on syphilitic as contradistinguished from that which arises from simple causes. In this distinction the author has established a new point in diagnosis, and one which will affect a material and important difference in treatment. Within the last few years the opinion, that syphilitic lesions of the osseous system in young children were of rare occurrence, has been generally accepted, and even now the statements of earlier writers to this effect exercises a large influence upon a large number of practitioners. The writer in his introductory, gives a brief resumé of the scanty literature of the subject, and then commences at once upon the relation of personal cases. Those mentioned in this work are twelve in number, and form an interesting and well recorded series. Upon the facts elucidated in the study of these cases the work is founded, aided by deductions drawn from cases reported by other writers, a recital of which follows those reported by the author.

The whole subject is carefully reviewed, and the work forms one of the very best additions that has recently been made to American Medical Literature. The author insists, and very justly too, that a careful distinction should be made between the osseous lesions arising in cachectic children, and those the result of syphilis, and cites several instances where cases have been reported as syphilitic, in which the history of syphilis was either entirely wanting, or very imperfectly made. The habit of jumping at conclusions is one which some able medical men have acquired, and from this course many mistakes in diagnosis have been made in these affections.

The treatment advised by the author is such as will meet with general approbation. The mixed treatment is employed in a large number of instances, that is, the combination of iodide of potassium and mercury in the same prescription. Good food, proper hygienic surroundings, cleanliness and tonics are matters which are to be insisted on as of the greatest importance.

The work is presented to the profession in an elegant form. Printed on thick tinted paper and handsomely bound, it is an ornament to any library, but its intrinsic value is of greater importance, and will be found of a high order.

